# Screencast-o-Matic

**DOI:** 10.5195/jmla.2021.1207

**Published:** 2021-04-01

**Authors:** Ann Glusker

**Affiliations:** 1 glusker@berkeley.edu, Sociology, Demography, & Quantitative Research Librarian, Doe Library, University of California Berkeley

## INTRODUCTION

Screencasting, defined as creating a video recording with or without audio of real-time actions and/or content on a computer screen, has been of interest to libraries for some time now. An excellent early guide by tech guru Greg Notess, *Screencasting for Libraries*, was released in 2012 [1]. Screencasting has been used mainly for library instruction and communications and has been a valuable tool for pedagogies involving microlearning (content delivered in small portions). Since the 2020 pandemic, however, screencasting has come into its own as librarians, instructors, and others seek avenues for connecting with their users in asynchronous and hands-on modes to improve online learning while libraries are closed. Screencasts of a library resource can be watched at any time, watched multiple times, and adapted to varying learning styles and needs. They can also demonstrate a reproducible path through a resource, including mouse movements, choices and locations of user options on a page, changing to other screens, etc.

There are many options for screencasting tools, both free platforms offering basic features and paid platforms with greater complexity and sophistication. Of the latter, Camtasia (a product of TechSmith) and Captivate (a product of Adobe) are the best-known. As these platforms each require a fairly substantial financial investment and a steeper learning curve (especially for individuals and small organizations), many screencasters are turning to free tools, which are evolving to add features to their basic versions. Some tools have come and gone: Screenr and Screentoaster are no longer available, and the very popular program Jing, while still available, has been bought by TechSmith and is now called TechSmith Capture. CloudApp is a well-reviewed option that has entered the scene more recently, but Screencast-o-Matic remains an excellent and user-friendly option. This review will examine its features, advantages, and disadvantages.

### Audience and evaluation

One of the most important features of Screencast-o-Matic is its ease of use, especially if you are familiar with the basics of programs such as YouTube and Zoom. Many users can create a usable video from scratch within half an hour of first using the tool. The audience for Screencast-o-Matic includes anyone with some familiarity with online tools who needs to create content such as brief tutorials of processes, demonstrations of software, visual cues to navigating websites, etc.

However, taking a step back, no tool is completely ideal for every user. It is important to evaluate why users want to use the tool and what type of product they hope to create. The list of questions below was developed by science librarian Olivia Bautista Sparks to assist with selecting screencasting software, and it appears here with answers for Screencast-o-Matic [2]. These questions can be used as a first step toward deciding whether a particular screencasting tool will work for basic video creation. (To this list I would add, is there a steep learning curve? As noted above, in the case of Screencast-o-Matic, the majority of people find it intuitive and easy to use.)

- Does the software require installation or is it browser-based? *Either*
- Does the software record audio during screen capture? *Yes*
- What types of files are created? *MP4, AVI, FLV, GIF*
- Does the software require a log in? *No, unless you want to save videos to an account*
- What is the maximum recording length? *Fifteen minutes (basic version)*
- What are the options for video hosting? Can you save your files and host it on your own server? Are your hosted files in the public stream? *Can save files, can host videos on Screencast-o-Matic site*
- How accessible is the recording menu? Are you able to pause your recording? *It is easy to record; yes, you can pause*
- Are you able to edit or annotate your recording? Are you able to upload your screencast to online video streaming sites (i.e., YouTube or Vimeo)? *Yes, yes*
- Are useable URLs provided? *Yes, using quick share*
- Are the embed codes provided or easily accessible? *Yes, after uploading video to Screencast-o-Matic*

### Major features

Screencast-o-Matic does what many other programs do, which is capture a series of actions and/or images on a screen. Most importantly, however, it enables the user to get up to speed easily and quickly with a minimum of technical expertise and without needing to sign up for an account or download anything. This is important for minimizing privacy concerns, or if a user does not have administrative rights to their own computer.

**Figure 1:**
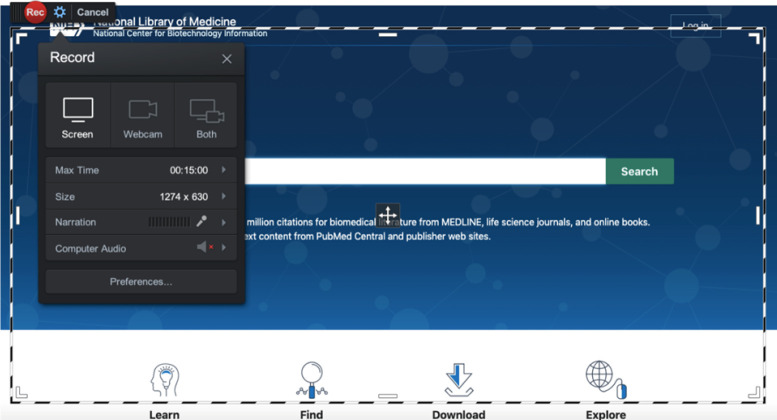
Recording options and frame

Making a video recording on Screencast-o-Matic is pretty much a matter of stopping and starting the process with controls that appear very similar to those on YouTube and other sites. Users can choose various options before recording, one of which is whether to capture content from the screen, webcam, or both. Figure 1 shows the red “Record” button at the upper left, the dotted line surrounding the frame that indicates the area to be captured, and the options for recording the screen, webcam, or both. Users can also set the program to highlight their cursor so that, when they click on something or move from one part of a page to another, the yellow ring around the cursor indicates the movements, making them more obvious. This can be very helpful for instructional videos. Recordings can be accompanied by narration into the user's microphone (the paid versions add the feature of being able to use audio from the computer as well).

**Figure 2:**
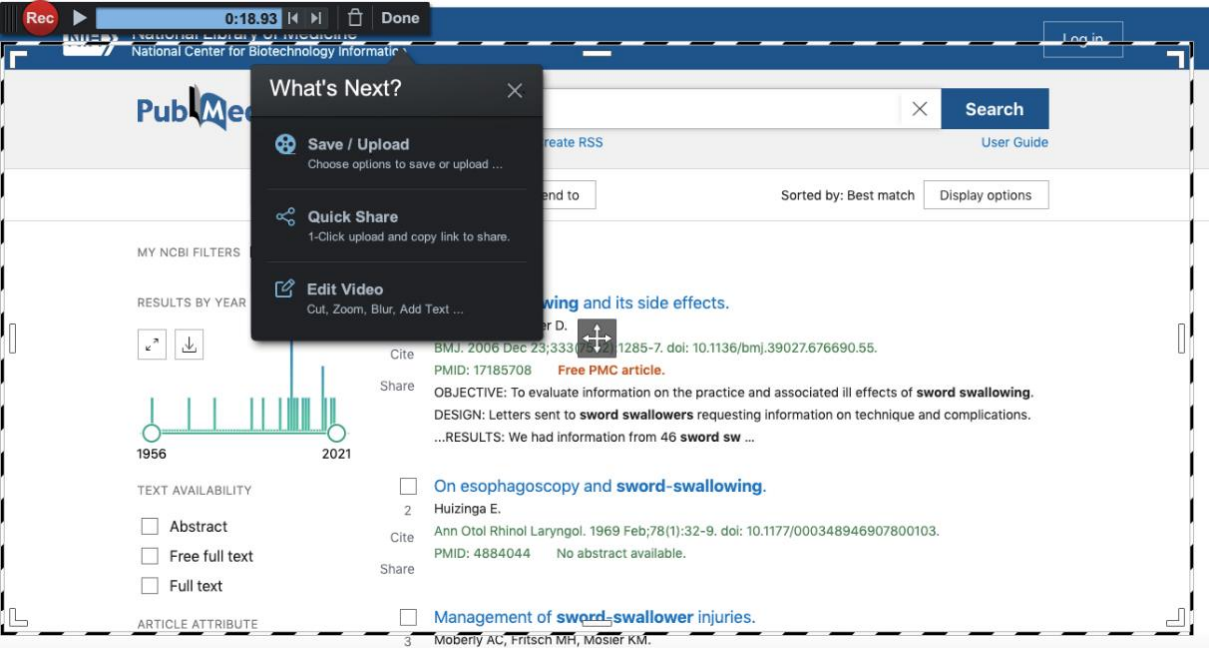
File saving/uploading, sharing, and editing options

**Figure 3:**
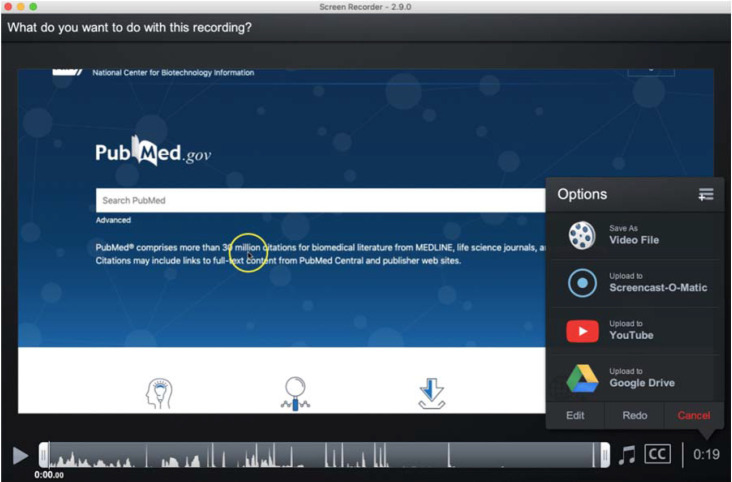
Detailed saving and uploading options

Once the video has been recorded, the user can either save it onto their own device in MP4, AVI, FLV, and/or GIF formats (there are apps for iOS and Android users) or directly upload it for sharing and hosting to the Screencast-o-Matic site (account required), YouTube, or Google Drive. Figure 2 shows the three steps involved in publishing (downloading) the completed video to the user's device. Figure 3 shows the more detailed options for saving and uploading the file, including the upload location options mentioned above. The paid options allow the user to enhance the created video using visual and sound editing capabilities (this can be useful if a narration track is recorded separately).

### Usability

While it has been mentioned several times that Screencast-o-Matic does not have a steep learning curve, that does not mean that there is no curve at all. Like any tool, it takes some getting used to. In addition, a disadvantage for learners is that the tool's website does not have an obvious search function. While it has extensive lists of tutorials, blog posts, and support documents, finding specific information can be challenging (this reviewer has ended up using Google's site search function too often).

On the other hand, there is abundant training and support available in general. The Blog, Tutorials, and Support pages each have articles and videos (of course!) to get users up to speed. Blog topics include “How Two Teachers Used Screencasts to Transform Education” and “7 Easy Editing Tricks for Social Media Videos.” Tutorial topics include a series of basic and advanced tutorials on editing, uploading, and other subjects, as well as technical topics such as using green screens. Support topics address IT questions for which users would request IT help, such as configuration, firewalls, and plan features. Screencast-o-Matic also offers a ticketing option—if you cannot solve your problem, you can submit a ticket to their support team.

Screencast-o-Matic works with Windows, Mac, and Linux operating systems.

### Integration

Screencast-o-Matic's “Integrations” page (screencast-o-matic.com/integrations) lists a wide range of more than thirty programs with which it can integrate. For some programs, like PowerPoint, the integration does not add much; the instructions say, basically, to “make a video recording of your presentation … and share it.” But for others, like Zoom, the integration creates something that is more than the sum of its parts. In this case (if you have a paid account), you can import Zoom meeting recordings into Screencast-o-Matic and then edit them [3]. Suggestions for editing include the following:

- mix and match videos and media;
- trim, crop, and cut segments of your videos;
- edit audio, replace narration, and add music;
- clean up or replace captions;
- highlight and zoom in/out on specific areas of your screen;
- add text, shape, blur, image, and video overlays, including from the Screencast-o-Matic stock media library; and
- add animations and transitions for effects [3].

Interestingly, a 2020 review of Screencast-o-Matic in *PCMagazine* [4], which is known for its reviews of technology and software, compared the program to SnagIt (another TechSmith product), which is a screen-capture program often used for snipping static images but which also has video capabilities. In this review, the disadvantages of Screencast-o-Matic were listed as not having OCR (optical character recognition) capabilities, being limited to the PNG format when exporting static images, and not being able to capture a scrolling screen (although there is a tutorial on the Screencast-o-Matic site called “Scrolling Screen Capture with PicPick,” so they know it is an issue). Perhaps SnagIt is worth a revisit, especially if any of these disadvantages of Screencast-o-Matic are deal breakers and if the cost of SnagIt is acceptable.

### Pricing

Screencast-o-Matic is available as a free resource and has robust features as described above. There are enhanced versions available at two levels of added features. For solo/individual users, the Deluxe version ($1.65/month, $19.80/year) adds the abilities to perform basic video editing, record audio from the computer, import narration and music, use scripted recordings, draw, zoom and create screenshots, create speech-to-text captions, use a green screen filter, and more. The Premier version ($4/month, $48/year) adds a custom video player and controls, a stock library, the option to share and collaborate with video hosting, a branded ad-free site, and 100 GB bandwidth, as well as other features. Team Plans follow these two bands of added features, starting at (for 10 computers) $9.50/month for the Deluxe and $17.50/month for the Premier.

### Value

An important question is where Screencast-o-Matic sits (or wants to sit) on the continuum of screencasting tools. As a high-functioning platform at an unbeatable price, Screencast-o-Matic is a major player at the lower end of cost and sophistication, but as users want to dive deeper into the full range of options for creating more refined products, they may want to transition into tools like Camtasia or Captivate, even considering the functionality of the added features of the paid versions of Screencast-o-Matic.

In the end, Screencast-o-Matic is an excellent tool for creating screencasts of solid quality, with its paid versions offering even more options for increasing the customization and sophistication of its products. It is easy to use and learn, has good documentation and support in its tutorials and blog posts, and offers flexibility for saving and sharing videos. This reviewer would strongly recommend it for creating high-quality screencasts with little fuss and learning curve and at no cost.
